# A hybrid concatenation packet aggregation approach for energy-aware disparate path routing in energy harvesting WSNs

**DOI:** 10.1038/s41598-026-47432-7

**Published:** 2026-04-14

**Authors:** N. Mookhambika, J. Raja, K. Srihari

**Affiliations:** 1 Department of computer science and Engineering, Sri Ramakrishna Engineering College, Vattamalai Palayam, NGGO Colony, Coimbatore, 641022 India; 2 Adhiparasakthi Engineering College, Melmaravathur, chennai, 603319, Tamilnadu India; 3 Department of Computer Science and Engineering , SNS College of Technology, Saravanampatti, Coimbatore, 641048 Tamil Nadu India

**Keywords:** Improved energy efficient disparate path routing, Concatenation packet aggregation algorithm, Engineering, Mathematics and computing

## Abstract

In a sensor network, a sensor node does not aggregate the data packets efficiently, since the packet is dropped for poor connection between the sensor nodes in the network. The sensor node location is varied in every area, so infrequent to share the information with its neighbor by the sender node. The communication is lost, and only received a minimum amount of packets for every communication, remaining packets are dropped. The node performs again packet transmission on the path makes the minimum residual energy level of each node. It increases the packet loss rate and energy consumption. Proposed IMPROVED ENERGY EFFICIENT DISPARATE PATH ROUTING (IEEDP) method is designed to achieve energy efficient packet aggregation along network environment. The position of the node is monitored clearly and then assign disparate path for routing. Concatenation Packet Aggregation algorithm is designed for a sensor network, it gathers the data packets without any loss for the transmission period from the source node to a destination node in a network environment, energy preservation is a sequence to increase the lifetime of the network by analyzing the residual Energy Effects on EH-WSN. It reduces packet loss rate and energy consumption.

## Introduction

The sensor network is created by spatially dispersed independent sensor nodes, which contain the capability of self-processing and self-aggregation for the reason of sensing and organizing data packets. Difficulties in sensor networks are that sensor nodes guard by their energy level. Because the sensor network is used for surveying on the extended durable sensor network, should depend on the straight to the remaining energy saving scheme. Specifically, residual energy is the combination of energy usage and initial energy level of all sensor nodes during the process ^1^. The sensor network nodes with the ability to extract energy level from the nearby nodes in the structure. Energy saving in sensor network uses various sources of energy, like a battery, wind, automatic environment changes, and temperature changes. Always given that energy level, and store it for expectations for energy usage ^2^.

However, it’s used in various uses, the consumed energy that relies on the network environment; it is not capable of energy usage for the nodes endless. The single difficulty of the network is the failure in some sensor nodes sequentially, that provides the packet loss and consumes more energy for packet transmission ^3^. Otherwise, as long as the residual energy is greater else similar to the energy used in regular, energy does not represent a restriction on the lifetime of the sensors in a network environment.

For that condition, the node works in a sustainable model. Considering the information that the wireless sensor network nodes save energy from the environmental source node regularly ^4^, smooth among this type of energy basis is frequently unrestricted, in various uses the available energy varies noticeably more time remaining to the change its network condition. Energy saving does not support for communication in any critical situation ^5^. Though, in various critical conditions like security uses, allowing for stable speed of the destination node is not appropriate because the packet receiving latency must assure the requested goal. Additionally,the activity of destination node with a regular mobility causes the unwanted wastage of energy level.

Consequently, Surrounded by the time slot that destination node contains minimum accessible data in its neighborhood to organize ^6^, it needs to improve the mobility sequence to preserve its energy level for a successive period with the option of the maximum level of the present data packet. Considering some unreasonable statement on their problem creation ^7^, to re-evaluate the issue by attractive into account the heterogeneity of communication process and energy saving features of nodes more than various time gaps.

The issues depend on the statement such varied position of the destination node in the network ^8^, and its packet moving space at every time period is placed when the entire data packet organization process. The network behavior is not entirely used from the sensible point of analysis. It supports to increase the network transmission rate by obtaining almost possible metrics ^9^. Additionally, the statement of remaining energy level must not always get together in sensible data packet aggregation, because the prototype of the energy usage of the energy resources is not predictable and change for its considering regularly network situation. Subsequently, from the potential of wireless sensor network designers, the energy saving resources that involve the network transmission rate obtained sequence to reduce the network cost in the sensible packet aggregation scheme ^10^.

The key contributions of this paper are summarized as introduced below:

1. **Energy-aware disparate path routing (IEEDP):** A routing architecture that chooses dynamically several energy-efficient disparate routes depending on node location, remaining energy and path stability. This would reduce the amount of packets lost due to a weak intermediate node and enhance the routing reliability.
2. **Concatenation-based packet aggregation mechanism:** A lightweight packet aggregation strategy that concatenates multiple packets sequentially before forwarding, reducing redundant transmissions and communication overhead compared with conventional in-network aggregation.
3. **Energy harvesting–aware routing decisions:** The proposed scheme incorporates energy harvesting nodes into routing decisions, allowing nodes with replenishable energy to participate more frequently in data forwarding and preventing energy hole formation.
4. **Performance improvement through simulation:** The simulation findings based on the NS2 platform indicate that the proposed IEEDP approach offers better packet delivery ratio, link stability, and network lifetime and less packet loss and power consumption in comparison with current routing schemes of CCR and WAR.

## Related works

Vyas et al. ^11^ propose a recent method is known as constant connections in Multicast Mesh and Energy Efficient communication have launched that conquer this issue by allowing for energy efficiency of the nodes as a choice standard to increase the connection steadiness. Its presentation is distinguished with the accepted mesh-based multicast communication method. The experimental output indicates the present SLIMMER scheme provides better performance such as packet transmission rate, and energy usage, under the situation of different quantity of nodes, speed, and execution period.

Archana et al. ^12^ presents analteration to the network environment of the cluster hierarchy Wireless Sensor Network, it separating groups into mini sectors. All clusters for its mini sector works like a hierarchy from a minimum quantity of nodes in thenetwork. Simulation output from the implement adjustment shows a major enhancement in minimization of energy usage, and also improves the network lifespan, it is distinguished with the usual grouping hierarchy sensor network. Otherwise,a little minimum for transmission rate and packet success ratio in the nodes of these networks is experimental. It guides to counsel with this process to construct the network, used for sensing secured cultivation and different networks with a minimum rate of packet transmission.

Yan et al. ^13^ present this issue and categorize, present communication method for Wireless Sensor Network into two groups considering to its direction toward either standardized or varied sensor network. It is additionally divided into constant and varied individually. To provide the summary of a routing method in every scheme by short their features, restriction and uses. Ultimately, a quantity of unlocking problems in energy effective method plan for the wireless network shows. This survey indicates the sequence to convene varied user needs. It should distinguish with the constant sensor network, a communication method for an unstable wireless sensor network, it is assured to execute better performance, such improved packet success rate, minimize energy usage, and higher energy efficiency,and construction cost.

Bhatti et al. ^14^ Furthermore, sensor nodes are fixed in a network environment, mountains and related difficulties part are not easy to measure of its energy usage problems. At present, the sensor network is designed as reasonable grids of standardized range, and packet organization by using energy effective communication. This present scheme is an improved form of VGDRA method that is grid depending communication except lacking the idea of calculating energy on the foundation of space between nodes. The sensory environment is separated into reasonable nodes and group header is selected between all nodes in sensor environment that works to handle all members in the group.

Zhang et al. ^15^ presents the essential thought is to separate issues into various type by the grouping of traffic insist vectors, and answer an issue by a most proper scheme, parameters are transmission rate, energy usage and execution time, equivalent to its type. It highlight the significance of minimizing execution time as a pivotal objective. The achievement of superior transmission rates and energy efficiency is coupled with a reduction in execution time, indicating the method’s efficiency in real-time or time-sensitive applications.

Guru et al. ^16^ present improving the existing convention, increasing the energy restriction and consequently. Considering the various uses, and essential of wireless sensor network, preparation of accurate packet gathering for declining, Medium Access Control with the details forwarding through has its particular suggestion. Sensor nodes the radio hardware are most strength consumption processes; for this part, strong management and appropriate coverage range are vital for communication. This RD-MAC is tense out additional to combine the backlog management method which carries the interims of the remaining time period, is used to position the briefest scheme in the network environment and the over protective of the responsibility move forwards among the network nodes. It estimates the performance of present scheme and reduces energy consumption.

Mallick et al. ^17^ present sequence to guarantee correct packet transmissionwith nodes, its caregivers, it meeting point on many receiver methods are used to increase the good organization and success rate of the packet is better. The present communicating method is used to process then decide the better target node else a transmitting a regardless of virtual node speed thus provided that enhanced structure of present method. In experimental output indicates present method provides energy efficient routing should distinguish with previous methods, this present scheme provides the maximum transmission rate in conditions of a number of packets accepted.

Singh et al. ^18^ presents a technique for energy efficient communication should depend on each particle swarm analyzation with the V-LEACH rule. presentation distinguishwith previous leach method indicates the present technique offers an improved characteristics is applied to reduce the energy indulgence in the packet sharing among sensor nodes and improves the sensor network lifespan, as well previous qualified characteristics of parameters such minimum packet latency, maximum transmission rate, and lesser energy usage in compared to previous leach rule.

Pantazis et al. ^19^ proposed energy efficient communicating methods are divided into different methods such as Network environment, packet transmission part, Topology Depending and trustworthy communication. The communicating techniques should go to the initial part, next it is divided to horizontal or Tree. Next communicating technique part is separated as Query-based else logical and nonlogical depending communication. Then communicating technique part for the next separation is Position depending else Mobility depending. Final routing technique part for the next separation is the quality of service depending or many route depending.Investigation of energy efficient communication method for a sensor network is obtained. This classification is proposed method improves the performance compared with previous methods.

Sasirekha et al. ^20^ Sequence to obtain a better presentation between the previous scheme, efficient communication technique called group sequence mobile agent communication is presented. It makes occupied utilize of the merits of uniformly low energy adaptive clustering hierarchy and energy well-organized assembly in sensor details scheme. The experimental output shows that the present method is better performance compared with various identical communication method, it provides energy efficient packet sharing.

Gina Martinez et al. ^21^ present path chosen technique which is furthermore regarded as the network overload of restricted ability for node battery, this technique is used to monitor every nodes, solar energy is used for energy harvesting of every nodes in network reducing the energy usage by decreasing the path cost considering the packet transmission, with the energy excess for charging to energy is wasted that are reduced by this scheme. In experimental result provides the maximum amount of energy is saved.

Although several routing protocols have been proposed for energy-efficient communication in Wireless Sensor Networks, most existing approaches focus either on multipath routing or in-network aggregation independently. Traditional multipath routing approaches enhance reliability yet can greatly raise communication overheads because of needless packet transmissions. In the same manner, regular methods of packet aggregation minimize the number of packets but notwithstanding, does not directly address node-energy heterogeneity or energy harvesting capabilities.

Conversely, the reconstruction of the proposed IEEDP framework combines energy specific disparate path routing and concatenation based packet aggregation. The routing is active and ensures the selection of the energy efficient nodes and the avoidance of unstable links but on the other hand, the aggregation mechanism of retransmission of packets is achieved by concatenating packets in a sequence. This combined design is the distinguishing aspect between the suggested approach and conventional multipath or aggregation schemes.

The recent literature has discussed numerous spaces of intelligent computational solutions as means of improving the performance, efficiency, and flexibility of contemporary communication, learning and optimization systems. Predictive routing Predictive routing mechanisms have been proposed to enhance transmission of data on highly dynamic vehicular networks where time-series based predictive models have been proposed to predict network conditions and provide more consistent vehicle-to-vehicle communication ^22^. Deterministic routing policies have also been proposed to make concurrent payment channels networks more reliable and efficient in terms of transaction reliability and efficiency as routing uncertainty is reduced and throughput is increased ^23^.

The advanced perception methods have been applied in robotic systems to allow manipulation of the objects in the environment with high skill and accuracy especially with the occlusion-aware grasping technique that makes use of the contextual scene perception to enhance the grasp detection performance ^24^. Besides all these proposals have been made, use of computational models have been suggested in the rapid trajectory analysis and mission planning scenarios of multi-asteroid exploration where an efficient analysis of electric sail propulsion to deep-space missions can be performed ^25^. Machine learning systems have found popularity in cybersecurity to mount harmful behaviors, such as predictive models that identify phishing programs prior to causing widespread harm ^26^.

Much attention has also attracted to optimization algorithms, and optimized particle swarm optimization methods with reverse learning techniques and adaptive neighbor adjustment has been proved to improve on performance on complex tasks scheduling problems in space surveillance networks ^27^. Also, the federated edge learning has also become the subject of resource management and excepted methods have been examined where the collective user scheduling and bandwidth allocation algorithms are developed in order to enhance the efficiency of storage and communication of distributed learning systems ^28^. Neural architecture search methods have also developed many improvements, with training-free methods effective to identify high-performing neural network architectures with minimal computational cost ^29^.

More recent studies have also concentrated on making decision-making structures more efficient by applying transformer models into reinforcement learning systems such that more efficient sequential decision analysis of complex systems can be made ^30^. On the other hand, communication technologies have been further increased using better coding and decoding schemes that maximize stopping criteria to enhance greater reliability and transmission effectiveness ^31^. Resource allocation schemes backed by reinforcement learning have been suggested to optimize the spectrum use and network performance of optical methods of wireless communication ^32^.

Also, satellite-terrestrial integrated networks have mission oriented scheduling technologies to facilitate collaborative communication and reconfigurability of network ^33^. Multimodal developments have also led to the use of methods of geometric matching to enhance cross-modal retrieval with the process of matching disparate data representations ^34^. Also in the energy sector, coordinated control mechanisms have been suggested wherein there has been a stable provision and management of offshore wind power transmission systems of offshore wind power by utilizing the high-voltage direct current technology ^35^. In addition, semantically-conscious methods of robotic grasping have been established to aid in manipulations of objects at category level by integrating semantic perception and the robotic control systems ^36^. Generally, these works demonstrate the high speed of development of intelligent algorithms and learning based structures to handle the complex problems in real world.

## Overview of proposed method

The wireless nodes do not efficiently organize data packets from its intermediate node because those intermediate nodes are only used for packet relaying from the source node to target node in the network. The position of the wireless node is varied, therefore communication between those nodes are performed sequentially. If there is any packet gets dropped, then finally receiver accept the only lesser size of packets during the communication period, and also residual data are dropped. Sensors are located in flat, occasionally vertical, which perform communication should consume more energy for node location alteration in the network structure. It obtains more packet drop rate and less energy usage.

Presenting the IMPROVED ENERGY EFFICIENT DISPARATE PATH ROUTING (IEEDP) technique is applied to providing the energy efficient data organization by destination node along the network environment. The location nodes are analyzed noticeably and also it allocates the disparate route for communication.The concatenation packet aggregation algorithm is constructed and used for this network.

**Fig. 1:**
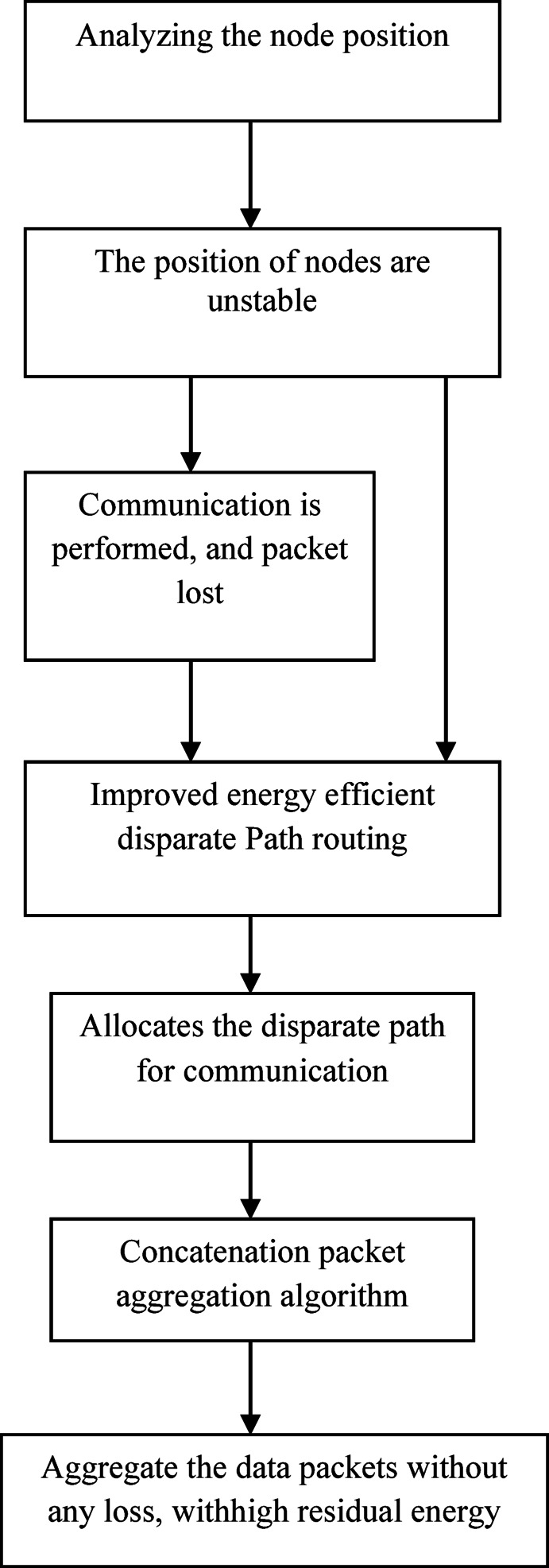
Proposed Flow Diagram on Improved Energy Efficient Disparate Path routing.

Figure 1 shows the proposed Improved Energy Efficient Disparate Path routing scheme, to analyze the every node position which is varied such as flat or horizontal. This node performed communication makes packet loss. The primary objective of the Improved Energy-Efficient Disparate Path routing scheme is to establish energy-efficient communication from a source node to a destination node. This is achieved by strategically allocating disparate routes for communication. A Concatenation Packet Aggregation algorithm has been carefully designed in order to achieve a smooth flow of data and minimal energy usage. This algorithm is developed to have the effect of aggregation of data packets which will reduce the chances of packet loss and will maximize the use of energy.

### Analysing the position of node with its coverage area (on the network)

Network node arrangement depends on the location such as flat or vertical for wireless nodes in a prearranged place. Acceptance node fixing isn’t conceivable in numerous uses considering the difficulties of network structure rule. Except it offers a direct method to analyze the node position, it is used to verify the different network structures, and to provide the efficient network structure for most extreme scope with least cover. Clarification for the issues of providing the maximum coverage in a destination with least number of intermediate sensor nodes for sensor structure. The node position arrangement is given for allocated routing. Additionally, possible node position arrangement is made between coverage possibility and the amount of arbitrarily placed sensor nodes in any network structure. Finally, a lot of sensor nodes are arranged in routing network based on possibility level. Where $EER$ energy efficient route, $Dr$ Disparate route, $CPa$ Concatenation Packet Aggregation.

1 $$EER=Dr+CPa$$

Restricted connection amongst monitoring and transmission limit, against the past connection amongst monitoring and transmission limit that indicates whether the transmission extends double for its monitoring range for the entire coverage range, involves the link in the network environment. The recent connection between transmission limit, with monitoring limit, is specified that indicates whether the lesser allocated path space among the sensor nodes is equivalent to monitoring level and whether the packet transmission double or triple monitoring limit subsequently full exposure in the network structure involve link establishment in the network environment. The simplification of condition for shortest routing path with maximum packet transmission range is normally double of entire coverage limit. Where $Np$ node position,$n*NL$ n number of nodes location analyzation, $Ps$ packet success rate.

2 $$Dr=NL\left(n\right)+Ps$$

Whether it is not able to deploy the sensors at the prearranged position after that different way to deploy the wireless nodes in the goal area is by casual exploitation. A little change in position where casual exploitation is applied for restricted area, unpredictable environment area, and information dropping area. To provide for random exploitation are cause more difficulties than predictable arrangement since for the random position of a sensor node in a wireless environment. Sensor chooses to go into the inactive condition according to precedence estimation depends on data packets organized from intermediate nodes. This minimizes the quantity of packets and, reject sightless end in the final goal area and given that complete sensing range. Where $C\left(A\right)$ coverage area, $Eu$ energy harvesting node, $Pd$ packet delivery rate.

3 $$NL\left(n\right)=n*NL$$

4 $$n=C\left(A\right)+En$$

5 $$NL\left(n\right)=C\left(A\right)+En*NL$$

Sensor networks play a crucial role in obtaining a maximum sensing area, yet merely achieving extensive coverage is insufficient. The creation of a highly interconnected network is necessary. In this respect, a linked network model is applied, in which particular sensor nodes are given the responsibility of transmitting packets. Each of these nodes is provided with the means of sharing packets either directly, or, when needed, it solicits the help of relaying sensor nodes in the network setting. The linked type of network also stipulates that the packets of data going through any sensor node must reach the receiver node. This entails a smooth flow of information and packet exchange between the interacting nodes until the last destination is attained. Despite several mechanisms undertaken to make sure the coverage is complete without any loss of packets; it is important to mention that the lowest area of coverage would result in the loss of packets in the process of communication. Finding the appropriate balance between full coverage and coverage range is vital to the achievement of the optimization of the entire communication process and preventing the loss of packets in sensor networks.

### Improved energy efficient disparate path routing

When the routing path finding the procedure for source node in the network, and path finding change the connectivity of nodes should cause difficulty. The sequence to minimize the difficulty for path finding, and also reduce the energy usage. The path finding in the particular area with the present node possibility of coverage is measured. Simply while this previous coverage limit does not suitable to discover the efficient node, to increase the node coverage area. Nextpath finding distance is based on the appearance of destination node with the reflection of the minimum distance routing path. The path finding approach is defined as the position of the link connecting the intermediate node to the destination node. Through all path finding procedure follows the restricted level of coverage range.Sometimes node loss its energy is called an energy hole, an Energy harvesting node with its *En* factor, it works and harvest energy from the environment. Practically consider the harvested energy is used to sensor nodes for transmitting, and receiving data packets, and also it is an insignificant part of energy need for routeestablishment process is reserved. Obviously, the energy need for path evaluation is numerous mission of importance lesser than that necessary for data packet transmission. Also, it maintains the more residual energy for processing. Where $Pl$ path loss, $Pd$ disparate path.

6 $$Pl=\left(Pl\right)>max(Pl)$$

7 $$Ps=min(Pl)+max(Pd)$$

Controlling the random movement of nodes is used to minimize the quantity of communication overhead during the transmission process. It verifies for any innovative potential minimum distance path than the present route cause the communication overhead. Destination node should start the communication route creation. on one occasion destination node obtain its place, communicating route is preserve using smaller quantityfor sharing data packets. The packet overload of evaluating time usage for particular packet transmission in the current path. The part of target node concerned in the communication procedureismodifiedsequence to manage the energy consumption. Consequently, subsequent to a specific time slot, intermediate nodes should inactive for the previous start; it should active and start the communication when the existing group of nodes is going to the immobile state. This goal to minimize the overhead for packet transmission. Arbitrary destination node progress has minimum collision as simply minimum nodes get changed along the communicating route. These efficient nodes are used for routing, whether there is any node get failed, to provide the energy efficient disparate path for further communication.

8 $$Pd= \left(Pd\right)<max(Pd)$$

The sensor nodes present in the network are motionless also their time slot is coordinated. Those sensor nodes are proficient of altering the routing path. A particular destination node is capable to organize data packet from various sender node at any arbitrary time period. Sensor nodes are identical in the environment it has energy with varied level. Originally, each the sensor nodes contain varied energy level. All nodes are an appeal to its connected timer arbitrarily. The node that timeouts the previous process gets chosen as routing, and find next neighbor for next time slot. Intermediate nodes are accepting the alerts from this chosen node terminate its execution time and choose this node as relaying. Because remaining intermediate nodes are used for routing, also maintain the coverage range of nodes in the path.

9 $$Dr=C\left(A\right)+En*NL+min(Pl)+max(Pd)$$

Destination node position details are maintained in routing packet that has x and y-axis level. Destination node accepting this beacon packet from the source node, this packet contains the information of node location. Destination node accepting the BEACON packet that verifies x and y-axis level of node details are available or not. If it equivalent to the destination node x and y-axis, then destination node needs to send a reply packet to the source node. The reply packets are moves toward to destination node with its next neighbor nodes available in routing path. Intermediate node loss packets for routing process are performed. The packet loss is made it rebroadcasting is carried this is avoided by using this energy efficient disparate path finding scheme.
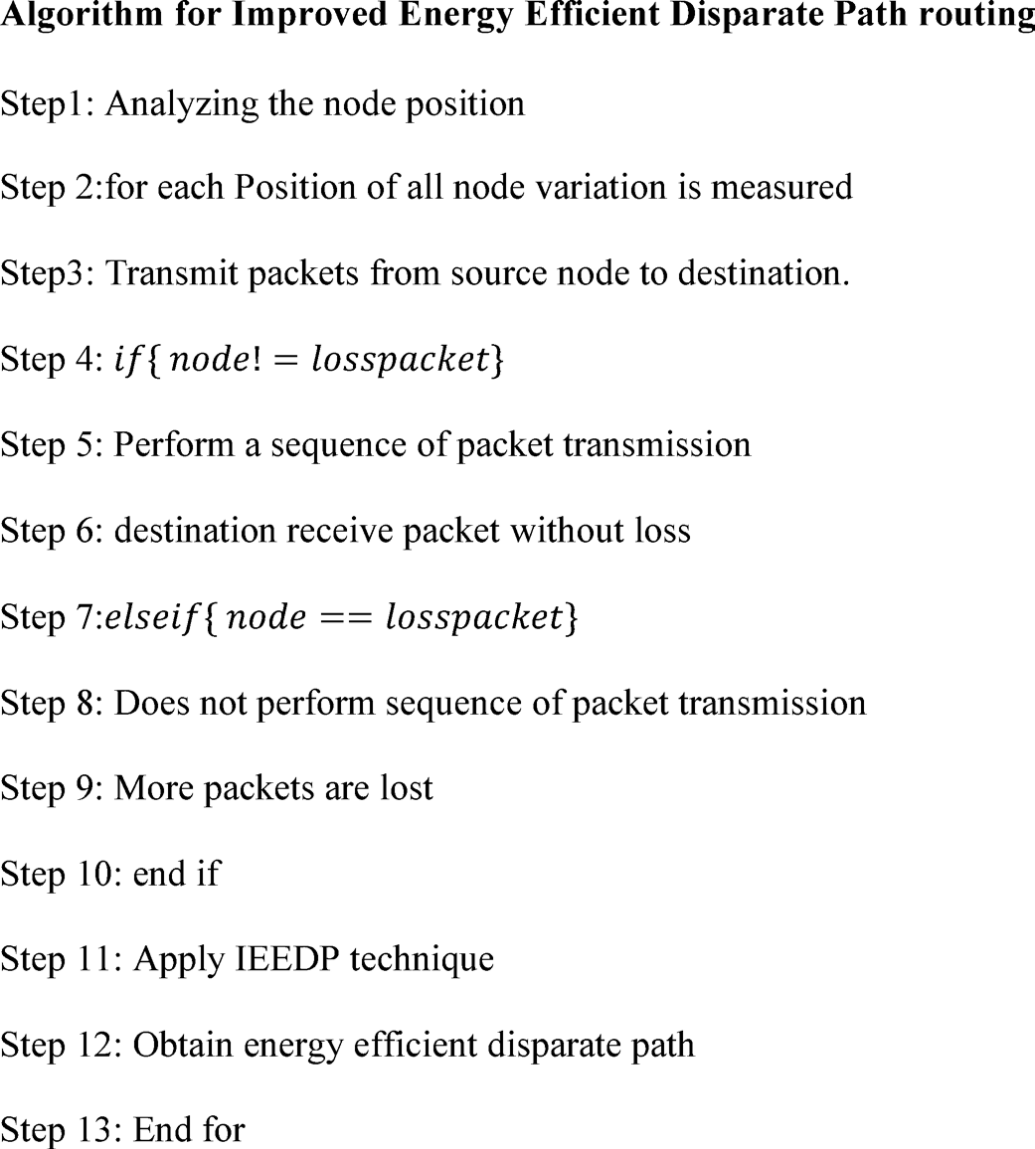


### Concatenation packet aggregation algorithm

Present scheme analyze the sensor nodes position to allocate the coverage range for communication. The distance from the source, and all intermediate nodes are analyzed. The packets are frequently collected by the destination node, by using this concatenation packet aggregation algorithm, which is constructed and applied to achieve energy efficient communication. The data packets are sequentially transmitted towards the destination node.

10 $$CPa=Seq(p1+p2+\dots +pn)$$

11 $$EER=\left\{C\left(A\right)+En*NL+min\left(Pl\right)+max\left(Pd\right)\right\}+Seq(p1+p2+\dots +pn)$$

Data packet is aggregated by destination without any disturbance, which indicates packet loss, and packet overload. It minimizes the packet loss at the current communicating node by usingonly energy efficient node so it avoids the error packet for packet transmission period. Packets aresent towards to destination in minimum distance path, so use the best intermediate node, it minimizes the packet overhead. Concatenation Data packet aggregation process carry on pending distinguished packet obtained to the destination node. This Improved Energy Efficient Disparate Path routing with Concatenation packet aggregation algorithm is used to provide efficient communication compared with normal energy efficient routing ^19^.
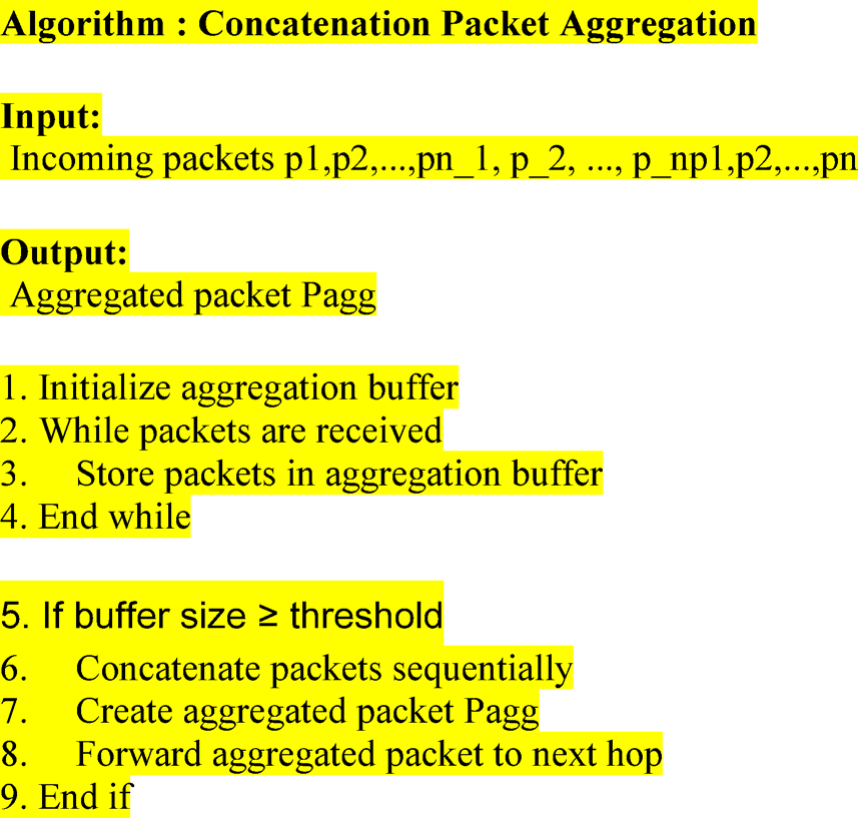


The sensor nodes will organize the data packets frequently by using this concatenation packet aggregation algorithm is constructed.

**Packet ID:** Packet ID has each and every sensor node information. It also contains node current place or location, such as vertical or flat in the network structure.

**Fig. 2:**
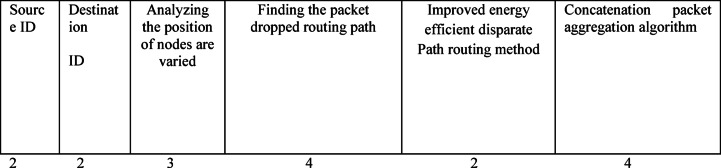
Proposed IEEDP Packet format.

In Fig. 2: the proposed IEEDP packet format is shown. Here the source and destination node ID field each should carries 2 bytes. The third one is Analyzing the position of nodes are varied in a network environment, it occupies 3 bytes. The source node finds the path based on node position as a located area such as x and y-axis in structure. In the fourth field occupies 4 bytes. Finding the packet dropped, routing path, in normal routing if intermediate node loss the packet, because those nodes are inefficient in nature. In fifth occupies 2 bytes, Improved Energy Efficient Disparate Path routing method, Allocates the disparate route for communication. The last field is a Concatenation Packet Aggregation algorithm, which takes 4 bytes.

**Algorithm 1:**
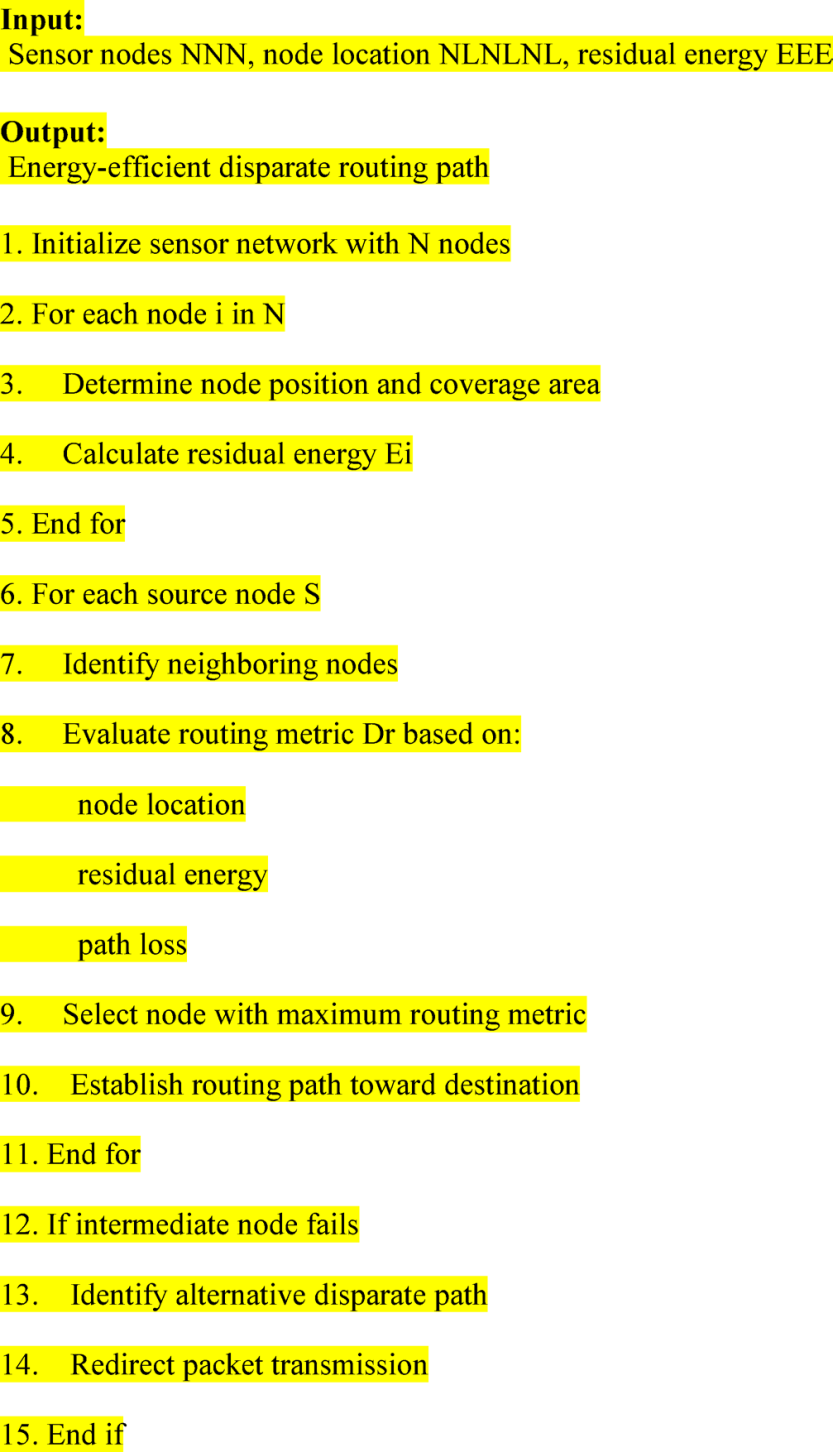
IEEDP Routing

## Performance evaluation

### A. Simulation model and parameters

The proposed Improved Energy-Efficient Disparate Path (IEEDP) scheme has been subjected to simulation using the Network Simulator tool (NS 2.34). All nodes in the simulation share a consistent transmission range of 250 m. To regulate the traffic rate and ensure a constant speed of packet transmission throughout the network, the Constant Bit Rate (CBR) mechanism is employed. This approach helps maintain a stable and controlled flow of data within the network. For the routing protocol, the Destination Sequence Distance Vector (DSDV) routing protocol is adopted. DSDV is selected to facilitate energy-efficient communication within the sensor network structure while mitigating the risk of packet loss. The application of DSDV is essential in the enhancement of the integrity and reliability of data transmission. Table 1 gives a detailed description of the simulation set up with critical and key values and settings as well as the setup to enable a detailed explanation of how the estimation process took place. This simulated framework will provide the real conditions of the proposed scheme of IEEDP performance in dynamic circumstances in the given network environment.

**Table 1:** Simulation Setup.

No. of nodes	100
Area Size	925 X 930
Mac	802.11 g
Radio Range	250 m
Simulation Time	34 ms
Traffic Source	CBR
Packet Size	512 bytes
Mobility Model	Random Way Point
Protocol	DSDV

**Fig. 3:**
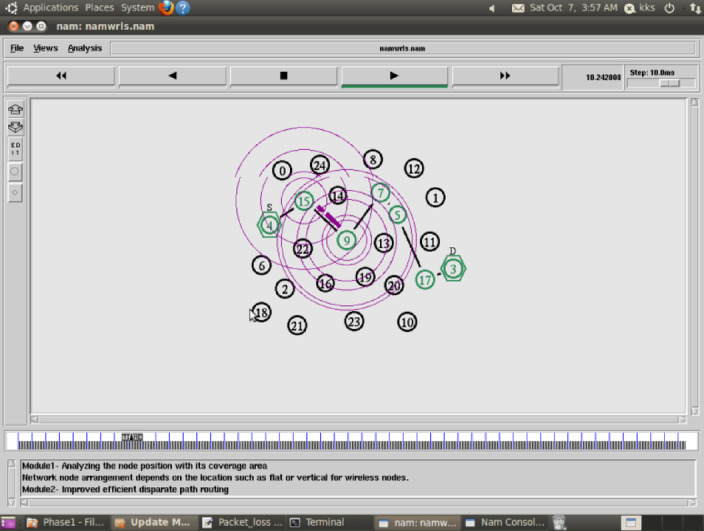
Proposed IEEDP Result.

**Simulation result:** Fig. 3 indicates that Improved Energy Efficient Disparate Path routing (IEEDP) technique resolves the energy efficient communication without packet loss is relative to current CCR ^20^, and WAR ^21^. IEEDP scheme builds Concatenation packet aggregation algorithm, it is the algorithm to use in assigning sequence of packet transmission towards the destination node, it uses minimum distance path routing. It reduces the rate of energy and packet loss.

**Performance analysis**: In simulation to examine the subsequent performance indicators with X graph in ns 2.34.

**Fig. 4:**
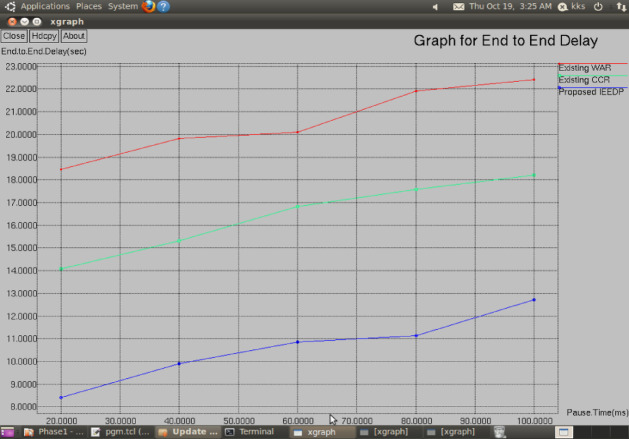
Graph for Pause Time (ms) versus End to End Delay.

Figure 4 shows how the end-to-end delay changes as a function of pause time. The proposed IEEDP technique has considerably reduced delay when in comparison to CCR and WAR. This is better off considering that IEEDP routing scheme chooses the energy efficient nodes of intermediate nodes whose connectivity is stable, and minimizes the retransmission of packets and routing overhead. Further, the concatenation packet aggregation mechanism minimizes the amount of packet transmissions thus minimizing total degree of delay.

$$EndtoEndDelay = EndTime{-}StartTime$$

**Fig. 5:**
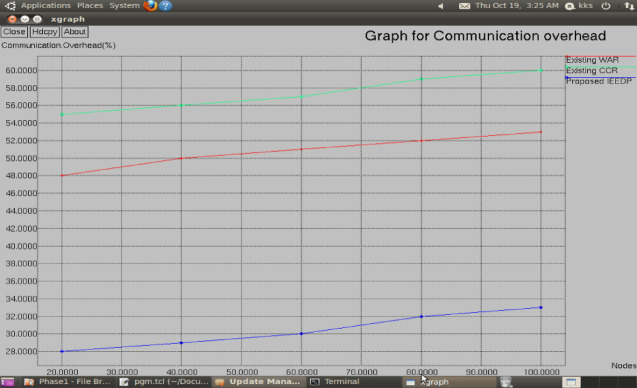
Graph for Nodes versus Communication overhead.

Figure 5 illustrates the communication overheads of varying networks. The overhead generated by the IEEDP technique is less than the current protocols since the unproductive nodes have been locked out during route structure. Also, the concatenation aggregation approach reduces unnecessary packet transmissions resulting in the formation of various packets into a single aggregated packet.

$$Communicationoverhead= (NumberofPacketLosses/Received)*100$$

**Fig. 6:**
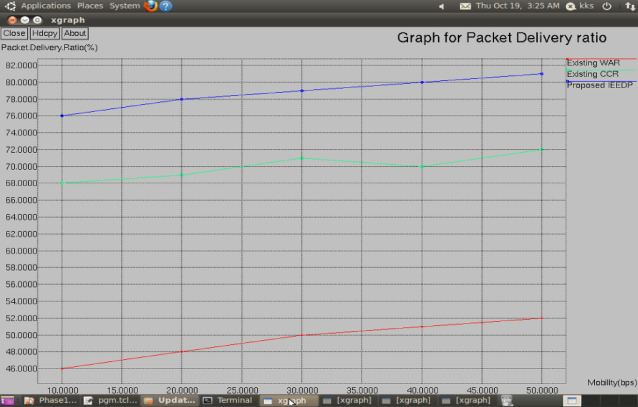
Graph for mobility (bps) vrsus Packet Delivery ratio.

**Packet delivery ratio:** Fig. 6 shows the Packet Delivery Ratio (PDR), which is obtained by dividing the number of packets received with the number of packets sent at a given speed. It is an interesting point to note that the velocity of nodes is not constant, and the simulation mobility is constant at 100 bits per second (bps). Figure 6 clearly shows that the IEEDP method outperforms the opposing approaches of deer walking, such as the WAR and CCR; the visual representation of the simulation results is that the IEEDP method is more effective in Packet Delivery Ratio and thus successful packet delivery as opposed to the two other methods.

$$PacketDeliveryRatio= (Numberofpacketreceived/Sent)*speed$$

**Fig. 7:**
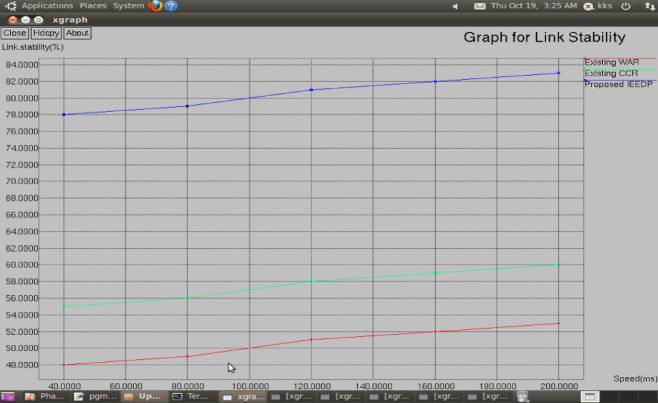
Graph for Speed (ms) versus Link Stability.

**Link stability:** Fig. 7 illustrates the Link Stability which is a critical measure that shows the strength of connections in a network. The Improved Energy-Efficient Disparate Path (IEEDP) routing algorithm has been developed to provide a greater stability in the links by actively evading weak linkage among the routers within the course of routing. The proposed IEEDP results in a significant increase in the Link Stability Ratio as compared to other solutions that are on existence like the Wireless Adaptive Routing (WAR) and Constant Congestion Routing (CCR). This higher Level of Stability of the Link is an indicator of the capacity of the IEEDP to develop better and more solid connections among the nodes, eliminating the risks created by poorly developed or fragile links. The visual approach of Fig. 7 confirms the superiority of the IEEDP approach over the stability of links by outlining the fact that it has much better ability to enhance the overall resilience and connection within the network relative to the WAR and CCR methodologies.

Figure 6 presents the packet delivery ratio under varying mobility conditions. IEEDP achieves higher PDR because the disparate routing mechanism identifies alternative routing paths when link instability occurs. This dynamic path adjustment improves reliability and ensures successful packet delivery.

$$Linkstability= weakconnection/overallconnection$$

**Fig. 8:**
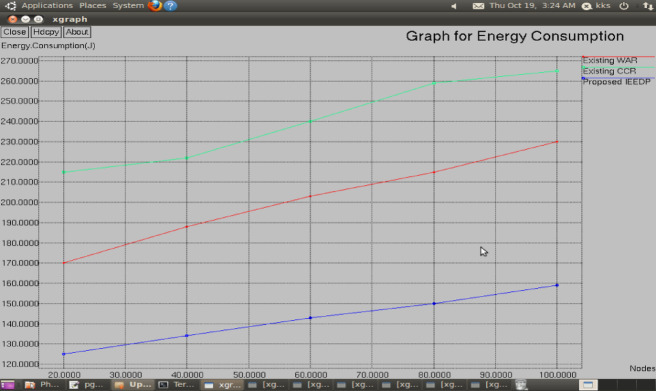
Graph for Nodes versus Energy Consumption.

**Energy consumption:** Fig. 8 shows energy consumption, how extended energy spends for communication, that means calculate energy consumption starting energy level to ending energy level. In proposed IEEDP method offer the energy-efficient routing path for sequence transmission of the packet; energy consumption is minimized compared to existing techniques.

$$EnergyConsumption= InitialEnergy-FinalEnergy$$

**Fig. 9:**
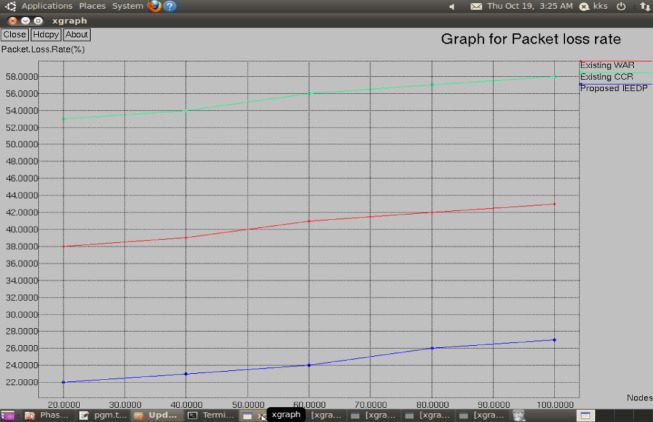
Graph for Nodes versus Packet loss.

**Packet loss:** Fig. 9 shows that the inefficient routing node that is rejected by using this Concatenation Packet Aggregation algorithm, it provides the concatenation of packet aggregation by destination along the efficient path. In proposed IEEDP method Packet loss is reduced compared to existing method WAR and CCR.

$$Packetloss=\left(Numberofpacket\frac{dropped}{Sent}\right)*100$$

**Fig. 10:**
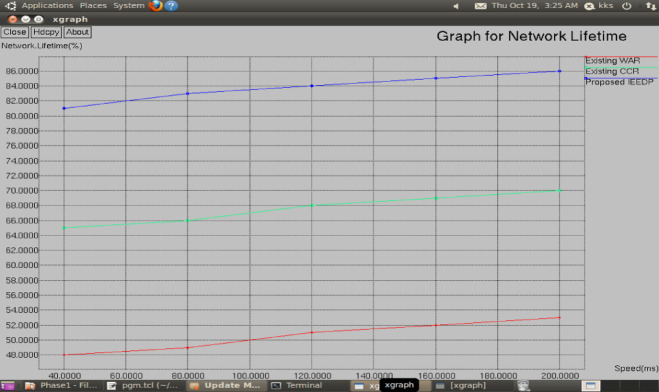
Graph for Speed versus Network Lifetime.

**Network lifetime:** Fig. 10 show that Lifetime of the network is evaluated by an overall process of the network, resource is taken like energy usage to obtain the best transmission in clustering, the Concatenation Packet Aggregation algorithm is used to provide an energy efficient path, it saves more residual energy and prolong its lifetime.

$$NetworkLifetime= lengthofenergyusage/overallenergy$$

The proposed IEEDP methodology is designed to support large-scale wireless sensor networks. The scalability of the system is achieved through three main aspects:

1. **Distributed routing decision:** The individual nodes then determine their adjacent nodes in relation to residual energy, the area covered as well as path loss. This decentralized decision making does not require centralized control and enables scaled routing mechanism to a large scale when the nodes are increased.
2. **Energy-aware node selection:** The routing paths are built by choosing nodes that have greater residual energy and energy harvesting capacity. This eliminates the problem of early node depletion and offers stable network connectivity despite the growing size of the network.
3. **Packet aggregation efficiency:** The concatenation packet aggregation algorithm reduces the total number of transmissions in the network. As the network size increases, aggregation helps control communication overhead and maintains efficient data transmission.

Therefore, the IEEDP framework can scale effectively in terms of:

Network size (number of nodes)

Network area coverage

Traffic load

Data generation rate

## Conclusion

This paper reported an Improved Energy Efficient Disparate Path Routing (IEEDP) model and a Concatenation Packet Aggregation algorithm on wireless sensor networks, which have the capability of energy harvesting. The offered solution examines the positions of the nodes, remaining energy, and features of coverage to determine the energy-efficient routing path of the source and destination nodes. In contrast to the traditional routing schemes, the IEEDP approach is dynamic in the selection of disparate routing paths, which also achieves packet aggregation to minimize communication overheads. The concatenation packet aggregation protocol consists of the combination of multiple packets into one transmission unit thus reducing retransmission as well as increasing the overall network effort. The performance of the proposed IEEDP method has been shown in the simulation based on NS-2 platform to be better than current routing protocols like CCR and WAR based on the ratio of packets delivered, stability of links, lifetime of the network, and energy usage. Moreover, the suggested routing scheme is useful in the minimization of packet dropping and overhead in communication in dynamic networks. Further research will be done on integration of smart routing schemes and detection method of abnormal energy consumption to enhance the routing efficiency and network life in large scale wireless sensor networks.

## Data Availability

The datasets used and/or analysed during the current study available from the corresponding author on reasonable request.
